# Effects of non-nutritive sucking combined with kangaroo care during blood sampling from the heel of newborns

**DOI:** 10.3389/fped.2026.1936904

**Published:** 2026-10-02

**Authors:** Xiao-Ping Shi, Ying Chen

**Affiliations:** Department of Clinical Laboratory, Huzhou Maternity & Child Health Care Hospital, Huzhou, Zhejiang Province, China

**Keywords:** application effects, blood sampling from the heel, kangaroo care, newborns, non-nutritive sucking

## Abstract

**Background:**

The quality of heel blood sampling is critical to the accuracy of neonatal disease screening. However, neonates inevitably experience pain during heel-lance procedures, and repeated painful stimuli may adversely affect neural development. This study aimed to evaluate the effectiveness of non-nutritive sucking combined with kangaroo care in improving procedural outcomes during heel blood sampling in newborns.

**Methods:**

This was a single-centre, non-randomised, quasi-experimental historical-control study. A total of 186 full-term newborns delivered vaginally and undergoing heel blood sampling for neonatal disease screening in the Department of Obstetrics at Huzhou Maternal and Child Health Hospital between July 2024 and June 2025 were included. According to the calendar period, newborns were included in a historical control group receiving routine care from July to December 2024 (*n* = 102) or an intervention group receiving non-nutritive sucking with a pacifier combined with kangaroo care from January to June 2025 (*n* = 84). Outcomes assessed included first-attempt success rate, blood drop diameter, bleeding time, and Neonatal Infant Pain Scale (NIPS) score. Adverse events such as hematoma, bleeding, and ecchymosis were also documented.

**Results:**

The intervention group demonstrated a significantly higher first-attempt success rate (97.62%) compared with the control group (83.33%; *P* < 0.05). The blood drop diameter in the intervention group (11.28 ± 2.03 mm) was greater than that in the control group (10.02 ± 1.85 mm; *P* < 0.05), while bleeding time was significantly shorter (2.48 ± 0.37 s vs. 3.51 ± 0.30 s; *P* < 0.05). The NIPS score during sampling was also markedly lower in the intervention group (3.36 ± 0.41) than in the control group (5.23 ± 0.68; *P* < 0.05). Additionally, the overall incidence of adverse puncture events was 10.71% in the intervention group, significantly lower than that the 22.55% observed in the control group (*P* < 0.05).

**Conclusion:**

The combination of non-nutritive sucking and kangaroo care was associated with favourable outcomes in neonatal heel-prick blood sampling, including higher first-attempt success, larger blood-drop diameter, shorter bleeding duration, lower procedural pain, and fewer puncture-related adverse events. These findings suggest that the combined intervention may be a feasible non-pharmacological approach for neonatal procedural pain management.

## Introduction

Newborn screening refers to the application of rapid, simple, and sensitive testing methods during the neonatal period to detect a range of congenital and hereditary disorders that threaten neonatal health and may result in physical and intellectual developmental impairments. This screening plays a vital role in reducing the burden of birth defects (1). The screening workflow includes blood specimen collection, specimen preparation and storage, sample transportation, laboratory testing, re-testing of positive cases, definitive diagnosis, and subsequent treatment. Among these steps, standardized blood specimen collection is closely linked to the overall quality of screening and is essential for ensuring its successful implementation (2). However, neonates inevitably experience painful stimuli during heel-prick blood sampling (3). Pain is a subjective sensation typically associated with actual or potential tissue injury. It may present as a localized symptom or as part of a systemic response. Previous studies have demonstrated that pain can exert both short-term and long-term adverse effects on neonates; repeated exposure to painful stimuli may alter brain structure and hormone levels, potentially leading to impairments in cognitive, motor, and neurodevelopmental outcomes (4, 5). Consequently, effective pain management is critically important for minimizing both immediate and long-term harm.

There is now broad consensus in the medical community that neonatal pain should be actively addressed. Among non-pharmacological analgesic interventions, non-nutritive sucking and kangaroo care are the most widely utilized (6). Non-nutritive sucking uses a pacifier in place of the maternal nipple to stimulate oral tactile receptors through rhythmic sucking, thereby increasing pain thresholds and promoting neonatal calmness (7). Kangaroo care, which involves skin-to-skin contact between the mother and the infant, is a simple and effective non-pharmacological method for alleviating neonatal pain. It emphasizes early and sustained skin-to-skin contact and aligns with the ideal practice of exclusive breastfeeding (8). Although both interventions have been investigated individually for neonatal procedural pain, evidence regarding their combined use during heel-prick blood sampling remains limited. Importantly, non-nutritive sucking and kangaroo care may act through partially different but complementary mechanisms: non-nutritive sucking provides oral sensory stimulation and behavioral regulation, whereas kangaroo care provides skin-to-skin contact, warmth, and maternal sensory input. Combining these approaches may therefore provide more comprehensive regulation of pain and distress during heel-prick procedures. Because procedural success and safety may also be influenced by the infant's behavioral state and tolerance of the procedure, first-attempt success, blood-drop diameter, bleeding duration, and local puncture-related adverse events were assessed alongside pain intensity.

Therefore, this study aims to evaluate the effectiveness of non-nutritive sucking combined with kangaroo care in reducing procedural pain and improving blood sampling-related outcomes during heel-prick blood collection in term neonates. We hypothesized that the combined intervention would reduce procedural pain and distress and improve the efficiency and safety of heel-prick blood sampling in term neonates.

## Methods

### Study design and setting

This was a single-centre, non-randomised, quasi-experimental historical-control (before-and-after) study conducted at Huzhou Maternal and Child Health Hospital. A total of 186 full-term neonates delivered vaginally, who underwent heel-prick blood sampling for newborn screening at the Department of Obstetrics, Huzhou Maternal and Child Health Hospital, between July 2024 and June 2025 and met the inclusion criteria, were enrolled in this study.

### Participants

The inclusion criteria were: (1) full-term neonates aged ≥3 days; and (2) an intact heel without local abnormalities that would preclude heel puncture. Exclusion criteria included: (1) congenital malformations, acute or chronic infections, or neurological signs or symptoms; (2) severe asphyxia, oxygen therapy, or mechanical ventilation; (3) severe cardiac, hepatic, or renal dysfunction, or respiratory insufficiency; and (4) use of analgesics within 48 h after birth. Group assignment was determined by calendar period rather than randomisation. The historical control group consisted of 102 neonates who received routine care between July and December 2024, whereas the intervention group included 84 neonates who received non-nutritive sucking combined with kangaroo care between January and June 2025. Thus, the study used a before-and-after comparison with a historical control period. No formal sample size or power calculation was performed because all eligible neonates meeting the predefined inclusion and exclusion criteria during the specified study periods were included.

### Intervention

The procedures in both groups were performed by the same team of neonatal nurses, all of whom had at least 5 years of experience in neonatal care. Before the study commenced, they received standardized training on heel-prick blood sampling and non-pharmacological pain interventions, and passed a competency assessment prior to participation. The staffing allocation remained fixed throughout the study period, with no personnel changes.

All neonates were kept in a quiet and awake state before blood collection. The procedure was uniformly scheduled within 2.0 h after the last feeding to avoid any influence of feeding status on pain responses. Three to five minutes before the heel lance, a warm towel was applied to the heel on the sampling side to dilate local blood vessels. During the procedure, neonates were placed in the supine position (control group) or in the prone kangaroo care position on the mother's chest (intervention group). The operator fully exposed the heel and used a disposable safety heel-lancet to puncture the medial or lateral aspect of the heel (avoiding the central and posterior parts). After puncture, the first drop of blood was discarded, and subsequent samples were collected. Following blood collection, sterile cotton swabs were used to apply pressure to the puncture site until bleeding ceased, and gentle soothing was provided. The specimens were immediately sealed and sent for laboratory testing.

For the control group, during blood collection, one nurse gently restrained the neonate's bilateral upper limbs and the contralateral lower limb, accompanied by light patting to soothe the infant. After blood collection, verbal comforting continued until the neonate became calm.

For the intervention group, in addition to the routine care, non-nutritive sucking (NNS) combined with kangaroo care (KC) was provided. A KC team consisting of trained paediatric nurses was established. On the three consecutive days (days 1–3) before blood collection, mothers and neonates received KC guidance: the mother's bed head was raised to a comfortable position, the neonate, wearing only a diaper, was placed prone on the mother's bare chest, with a warm blanket covering the back. Mothers were encouraged to talk softly, stroke the neonate's skin, or kiss the neonate. Daily KC sessions lasted at least 60 min. All mothers in the intervention group completed the full 3-day KC protocol (adherence was verified daily by nursing logs, ensuring no protocol deviations). On the third day, after 30 min of skin-to-skin contact during the ongoing KC session, the neonate remained in the prone KC position on the mother's chest, and the heel-prick blood collection was performed directly in this position by the nurse, to assess procedural pain while the neonate was receiving KC.

For the NNS intervention, one minute before the start of the blood collection procedure, a pacifier of uniform specification was placed in the neonate's mouth by the nurse to induce non-nutritive sucking. During the heel lance, the nurse continuously observed the neonate's sucking rhythm; if sucking ceased for more than 3 s, the nurse gently stroked the neonate's cheek to stimulate the sucking reflex and maintain effective sucking. The pacifier remained in place throughout the heel-prick procedure until hemostasis was achieved and the neonate was calm and stable, after which it was removed.

### Outcome measures

Sampling-related indicators were recorded, including first-attempt puncture success rate, blood drop diameter, and bleeding duration. Pain scores were assessed at five time points: 20 min before sampling, during sampling, and at 5, 10, and 20 min after sampling. Pain was evaluated on-site by a designated nurse using the Neonatal Infant Pain Scale (NIPS), a multidimensional tool for assessing procedural pain in preterm and full-term infants (birth to 6 weeks), including facial expression, crying, breathing pattern, arm movements, leg movements, and state of arousal (9). Scores range from 0 to 7, with higher scores indicating more severe pain. Local adverse reactions such as hematoma, bleeding, and ecchymosis at the puncture site were also observed and recorded.

### Data collection

During the study, the nurse recorded in real time the initiation time, total duration, and changes in sucking frequency of NNS. If vigorous crying caused the pacifier to fall out or sucking to be interrupted, it was considered a protocol deviation and noted in the original records.

Throughout the study, the team leader checked the nursing implementation logs daily to ensure that the two groups were consistent in terms of blood collection timing, lancet specifications, warm compress duration, operators, and operation techniques. All KC and NNS procedures in the intervention group were recorded with start and end times, as well as neonatal responses. No protocol deviations due to intervention interruption or neonatal intolerance occurred during the study period.

### Statistical analysis

Data were analyzed using SPSS version 20.0. Categorical variables were expressed as frequencies (n) and percentages (%) and compared between groups using the *χ*^2^ test. Continuous variables conforming to a normal distribution were presented as mean ± standard deviation $\left(\bar{x}\pm s\right)$ and compared using independent-samples *t*-tests and repeated-measures ANOVA as appropriate. Repeated-measures ANOVA was used to assess changes in NIPS scores over time, with group, time, and group × time interaction effects evaluated. Between-group comparisons were additionally performed at individual time points. A *P* value <0.05 was considered statistically significant.

### Ethical considerations

This study was reviewed and approved by the Ethics Committee of Huzhou Maternal and Child Health Hospital (No. 2021-R-001). Written informed consent was obtained from the parents or legal guardians of all participating newborns prior to enrollment. All procedures were conducted in accordance with the ethical standards of the institutional research committee and with the 1964 Helsinki Declaration and its later amendments.

## Results

### Baseline characteristics of newborns

The baseline characteristics of the neonates in the two groups were comparable. No statistically significant differences were detected in gestational age at birth, birth weight, or breastfeeding frequency (*P* > 0.05), indicating that the groups were well balanced and suitable for comparative analysis (Table 1).

**Table 1:** Comparison of baseline characteristics between the two groups of neonates.

Groups	*n*	Gestational age at birth (weeks)	Birth weight (g)	Breastfeeding frequency (times)
Control group	102	39.64 ± 2.52	3,359.07 ± 412.46	20.37 ± 2.85
Intervention group	84	39.58 ± 2.35	3,286.94 ± 379.57	21.06 ± 3.01
*t*		0.167	1.230	1.602
*P*		0.868	0.220	0.111

### Blood sampling–related indicators

In the intervention group, 82 neonates achieved successful blood collection with a single heel lance, corresponding to a success rate of 97.62%, which was significantly higher than the 83.33% (85 cases) observed in the control group (*χ*^2^ = 10.250, *P* = 0.001; OR = 8.200, 95% CI: 1.837–36.612). In addition, the intervention group demonstrated a significantly larger blood drop diameter (11.28 ± 2.03 mm vs. 10.02 ± 1.85 mm) and a markedly shorter bleeding time (2.48 ± 0.37 s vs. 3.51 ± 0.30 s) compared with the control group, with all differences reaching statistical significance (*P* < 0.05) (Figure 1).

**Figure 1:**
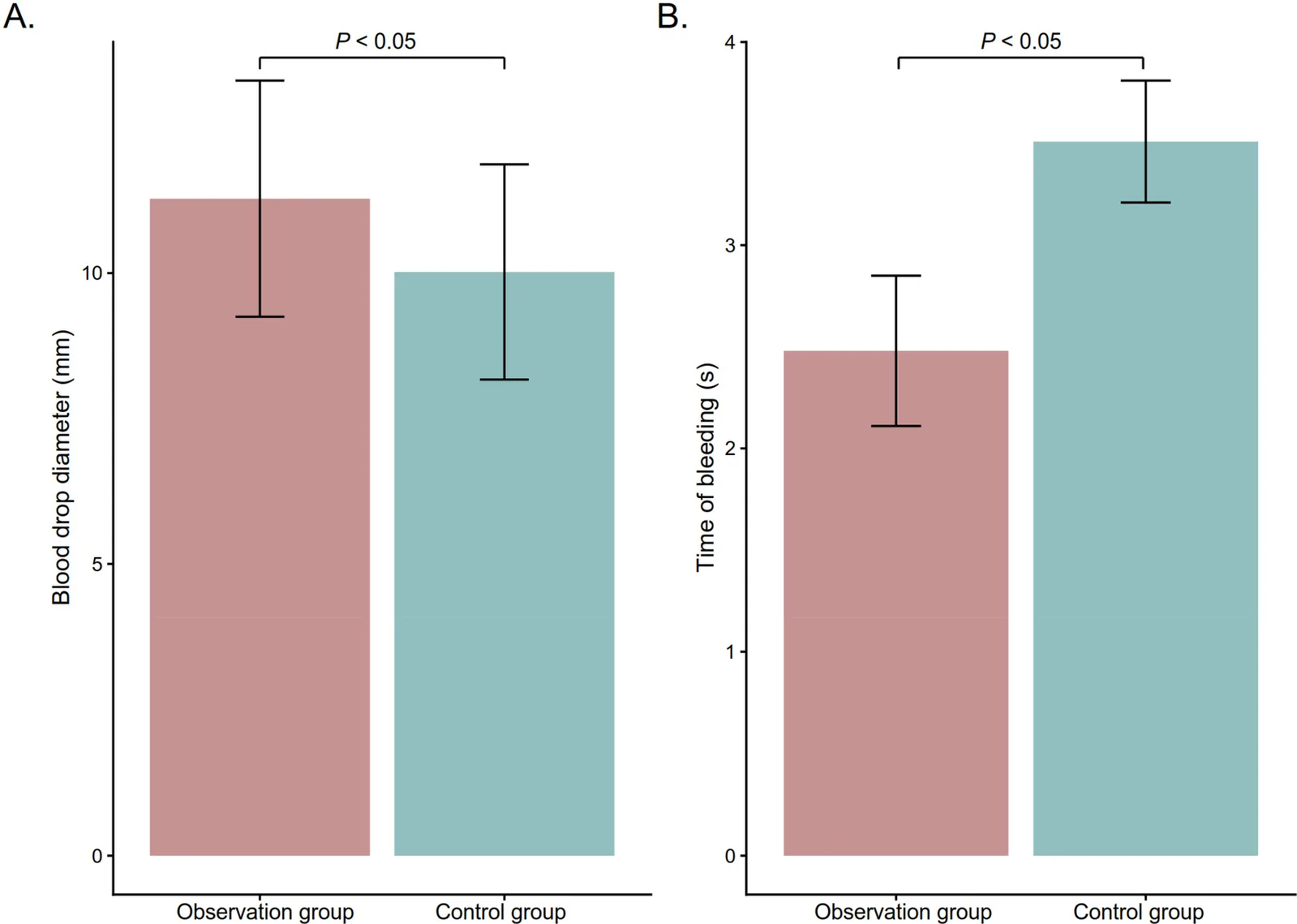
Comparison of blood drop diameter and bleeding time between the two groups. **(A)** Blood drop diameter (mm). **(B)** Time of bleeding (s).

### Pain responses

Repeated-measures ANOVA was performed to compare the neonatal NIPS scores between the two groups. The results revealed significant main effects of group (*F* = 15.902, *P* < 0.001) and time (*F* = 47.648, *P* < 0.001), as well as a significant group-by-time interaction (*F* = 8.233, *P* < 0.001), indicating that the trajectories of pain scores over time differed significantly between the two groups. Further analysis of between-group differences at each time point showed that there were no statistically significant differences in NIPS scores between the two groups at 20 min before sampling or at 5, 10, and 20 min after sampling (*P* > 0.05) (Table 2). However, the NIPS score recorded during blood sampling was significantly lower in the intervention group than in the control group (*P* < 0.05). The temporal changes in mean NIPS scores in the two groups are also illustrated in Figure 2, with error bars representing standard deviations.

**Figure 2:**
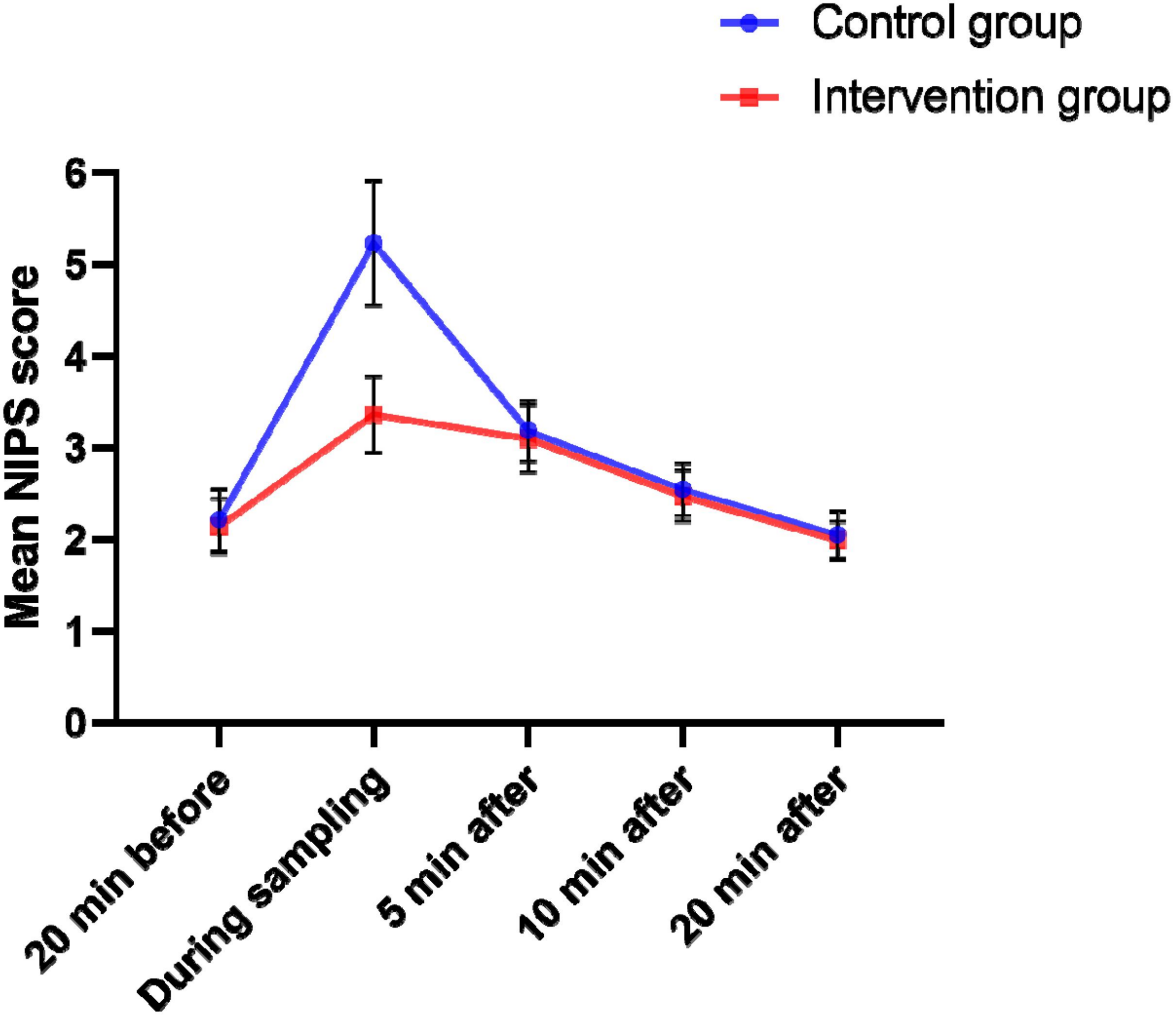
Temporal changes in NIPS scores during heel lance blood sampling. Data are presented as mean ± SD.

**Table 2:** Comparison of heel lance–related pain responses between the two groups.

Groups	20 min before sampling	During blood sampling	5 min after sampling	10 min after sampling	20 min after sampling
Control group	2.21 ± 0.34	5.23 ± 0.68	3.18 ± 0.33	2.54 ± 0.29	2.05 ± 0.26
Intervention group	2.14 ± 0.30	3.36 ± 0.41	3.10 ± 0.37	2.47 ± 0.28	1.99 ± 0.20
*t*	1.47	22.11	1.56	1.66	1.73
*P*	0.14	<0.01	0.12	0.10	0.089
*F*_group_	15.902, *P* < 0.001				
*F*_time_	47.648, *P* < 0.001				
*F*_interaction_	8.233, *P* < 0.001				

*F*_group_ = 15.902; *F*_time_ = 47.648; *F*_interaction_ = 8.233; all *P* < 0.001.

### Stick-related adverse events

In the intervention group, 3 cases of hematoma, 5 cases of bleeding, and 1 case of ecchymosis were reported. In contrast, the control group experienced 8 cases of hematoma, 12 cases of bleeding, and 3 cases of ecchymosis. The overall incidence of stick-related adverse events in the intervention group was 10.71%, markedly lower than the 22.55% observed in the control group. This difference was statistically significant (*χ*^2^ = 4.53, *P* = 0.03) (Table 3).

**Table 3:** Comparison of puncture-related adverse events between the two groups.

Groups	Hematoma	Bleeding	Ecchymosis	Overall events
Control group	8	12	3	23
Observation group	3	5	1	9
*χ* ^2^	1.511	7.874	0.671	4.529
*P*	0.219	0.171	0.413	0.033
*OR (*95%CI)	0.435 (0.112–1.695)	0.475 (0.160–1.406)	0.398 (0.041–3.894)	0.412 (0.179–0.948)

## Discussion

Heel lance blood sampling is routinely performed in term newborns for the early screening of hereditary and metabolic disorders. The success of this procedure depends on several factors, including the blood collection device, the infant's posture, environmental temperature, and the nursing interventions implemented before and during the procedure (10, 11). Given that heel lance is an invasive and often painful procedure, appropriate pain management strategies are essential not only to reduce neonatal distress but also to enhance procedural efficiency and improve the quality of blood samples obtained. Among the various non-pharmacological approaches available, non-nutritive sucking and kangaroo care have been widely recognized as simple, safe, and effective nursing interventions.

Non-nutritive sucking (NNS) involves placing a pacifier in the newborn's mouth to induce rhythmic sucking movements. This action provides rhythmic oral sensory stimulation that may modulate nociceptive transmission and attenuate pain perception (12). In addition, NNS has been shown to stabilize vital signs and improve behavioral state regulation, providing newborns with a greater sense of comfort and security during stressful procedures (13). Kangaroo care—defined as skin-to-skin contact between the newborn and the mother—has been shown to alleviate neonatal pain, reduce crying, and promote physiological stability through continuous skin-to-skin and multisensory stimulation (14). The analgesic effect of kangaroo care may involve modulation of sensory and stress-regulatory pathways (15). Because NNS provides oral sensory input whereas kangaroo care provides sustained tactile, thermal, and multisensory stimulation, their effects may be complementary when applied concurrently.

In the present study, the combined use of non-nutritive sucking and kangaroo care was associated with favourable outcomes across multiple aspects of the heel lance procedure. First, the intervention group showed a significantly higher first-attempt stick success rate than the control group. Achieving successful puncture on the first attempt is critical in neonatal blood sampling, as repeated attempts not only cause additional pain and distress but also increase the risk of local tissue injury. The higher first-attempt success rate observed in the intervention group may reflect better tolerance of the procedure, although the underlying mechanisms could not be directly determined in this study.

Second, the intervention group had a larger blood-drop diameter and shorter bleeding duration than the control group. A larger blood drop ensures adequate sample volume, which is essential for accurate newborn screening. These findings may indicate more efficient blood collection and may help reduce the need for repeated procedures, although the mechanisms underlying these procedural differences cannot be determined from the present study.

Third, the intervention group exhibited significantly lower NIPS scores during blood collection, indicating lower observed procedural pain during blood sampling in the intervention group. This finding is consistent with the established analgesic effects of these non-pharmacological interventions. The results reinforce the importance of implementing non-pharmacological pain management strategies in routine newborn procedures and support the potential complementary benefits of using these interventions concurrently.

Furthermore, the intervention significantly reduced the incidence of stick-related adverse events, including hematoma, bleeding, and ecchymosis. In this study, the total adverse event rate in the intervention group was less than half that of the control group. This finding may be clinically relevant because puncture-related adverse events can increase infant discomfort and may contribute to delayed local recovery. The lower event rate may be related to the observed differences in procedural success and tolerance, although causality cannot be established from the present study.

Our findings are consistent with those reported by Akbari et al. (7) and Wang et al. (16), who similarly observed that non-nutritive sucking and kangaroo care effectively reduce neonatal procedural pain and enhance the quality of blood sampling. Taken together, the results of this study and previous evidence suggest that combining these two interventions may provide a more comprehensive approach to procedural pain management than either intervention alone. Because the present study did not include separate kangaroo-care-only and non-nutritive-sucking-only groups, whether the combined intervention has a synergistic effect or is superior to either intervention alone cannot be determined. From a clinical perspective, this approach is particularly valuable because it is simple to implement, non-invasive, cost-free, and highly acceptable to both caregivers and parents. However, further prospective randomised studies are needed before routine clinical implementation can be recommended.

### Strengths and limitations

A strength of this study is that it evaluated not only neonatal procedural pain but also several blood-sampling-related outcomes, including first-attempt success, blood-drop diameter, bleeding duration, and puncture-related adverse events. By considering both neonatal comfort and procedural performance, the study provides a more comprehensive assessment of the potential clinical value of non-pharmacological care during heel-stick blood sampling.

Several limitations should be considered. First, this was a single-centre, non-randomised, quasi-experimental study using sequential calendar periods as a historical control; therefore, temporal and unmeasured confounding cannot be excluded, and the observed associations should not be interpreted as definitive causal effects. Second, the absence of separate kangaroo-care-only and non-nutritive-sucking-only groups precluded evaluation of their individual effects or any synergistic effect of the combination. Third, NIPS assessors were not blinded to group assignment, so observer bias cannot be excluded. Fourth, only term neonates meeting the specified eligibility criteria were included, and longer-term or repeated-procedure outcomes were not assessed, limiting the generalisability and scope of the findings. No additional potentially influential baseline or procedural variables were available beyond those reported. Future prospective randomised studies with appropriate comparison groups and longer follow-up are warranted.

## Conclusion

In summary, in this single-centre, non-randomised historical-control study, non-nutritive sucking combined with kangaroo care was associated with lower procedural pain and favourable blood-sampling outcomes in term neonates undergoing heel-stick blood collection. Compared with the historical control group, the intervention group showed higher first-attempt success, larger blood-drop diameter, shorter bleeding duration, and fewer puncture-related adverse events. These findings suggest that the combined non-pharmacological approach may be a feasible option for neonatal procedural pain management. However, because group assignment was determined by calendar period rather than randomisation, causal effects cannot be established, and further prospective randomised studies are needed to confirm these findings and determine the clinical value of the combined intervention.

## Data Availability

The raw data supporting the conclusions of this article will be made available by the authors, without undue reservation.

## References

[1] Therrell BL Padilla CD. Newborn screening in the developing countries. Curr Opin Pediatr. (2018) 30:734–9. 10.1097/MOP.0000000000000683 30124582

[2] Jiang H Yang R Dong A Wu B Zhao Z. Progress of newborn screening in China. Zhejiang Da Xue Xue Bao Yi Xue Ban. (2023) 52:673–82. 10.3724/zdxbyxb-2023-0467 38115737 PMC10764191

[3] Fitzgerald M. What do we really know about newborn infant pain? Exp Physiol. (2015) 100:1451–7. 10.1113/EP085134 26446174

[4] Cheong JLY Burnett AC Treyvaud K Spittle AJ. Early environment and long-term outcomes of preterm infants. J Neural Transm (Vienna). (2020) 127:1–8. 10.1007/s00702-019-02121-w 31863172

[5] Gokulu G Bilgen H Ozdemir H Sarioz A Memisoglu A Gucuyener K. Comparative heel stick study showed that newborn infants who had undergone repeated painful procedures showed increased short-term pain responses. Acta Paediatr. (2016) 105:e520–5. 10.1111/apa.13557 27557529

[6] Pillai Riddell RR Bucsea O Shiff I Chow C Gennis HG Badovinac S. Non-pharmacological management of infant and young child procedural pain. Cochrane Database Syst Rev. (2023) 6:Cd006275. 10.1002/14651858.CD006275.pub4 37314064 PMC10265939

[7] Akbari N Mutlu B Nadali J. Effect of non-nutritive sucking during heel-stick procedure in pain management of term infants in the neonatal intensive care unit: a systematic review and meta-analysis. Curr Pediatr Rev. (2022) 19:90–8. 10.2174/1573396318666220410225908 35410609

[8] Sharma H Ruikar M. Kangaroo mother care (KMC) for procedural pain in infants: a meta-analysis from the current evidence of randomized control trials and cross-over trials. J Family Med Prim Care. (2022) 11:1250–6. 10.4103/jfmpc.jfmpc_1383_21 35516684 PMC9067180

[9] Bellieni CV. Neonatal Infant Pain Scale in assessing pain and pain relief for newborn male circumcision. Int J Impot Res. (2023) 35:282–5. 10.1038/s41443-022-00551-x 35352017

[10] Anne RP Rahiman EA. Analgesic utility of automatic lancing device for heel prick in neonates: a systematic review and meta-analysis. Pain Manag. (2024) 14:375–83. 10.1080/17581869.2024.2368451 38940479 PMC11485952

[11] Baharlooei F Marofi M Abdeyazdan Z. Effect of environmental and behavioral interventions on pain intensity in preterm infants for heel prick blood sampling in the neonatal intensive care unit. Iran J Nurs Midwifery Res. (2017) 22:388–91. 10.4103/ijnmr.IJNMR_176_15 29033995 PMC5637149

[12] Feştilă D Ghergie M Muntean A Matiz D Şerb Nescu A. Suckling and non-nutritive sucking habit: what should we know? Clujul Med. (2014) 87:11–4. 10.15386/cjm.2014.8872.871.df1mg2 PMC4462418 26527989

[13] Gibbins S Stevens B. Mechanisms of sucrose and non-nutritive sucking in procedural pain management in infants. Pain Res Manag. (2001) 6:21–8. 10.1155/2001/376819 11854758

[14] Shukla VV Bansal S Nimbalkar A Chapla A Phatak A Patel D. Pain control interventions in preterm neonates: a randomized controlled trial. Indian Pediatr. (2018) 55:292–6. 10.1007/s13312-018-1270-z 29428919

[15] Mooney-Leber SM Brummelte S. Neonatal pain and reduced maternal care: early-life stressors interacting to impact brain and behavioral development. Neuroscience. (2017) 342:21–36. 10.1016/j.neuroscience.2016.05.001 27167085

[16] Wang F Zhang Q Ni ZH Lv HT. Effects of kangaroo care on pain relief in premature infants during painful procedures: a meta-analysis. J Spec Pediatr Nurs. (2022) 27:e12390. 10.1111/jspn.12390 35859291

